# Supplementary material for: A HSV1 mutant leads to an attenuated phenotype and induces immunity with a protective effect
Source: PLoS Pathog. 2020 Aug 10;16(8):e1008703. doi: 10.1371/journal.ppat.1008703 (PMC7440667; doi:10.1371/journal.ppat.1008703)
Supplement: S1 Text — Supplement to Fig 1 to display the mutated sequencing details of the Us11 gene of the M6 strain. (DOCX) [file ppat.1008703.s001.docx]

**S1 Text. The DNA sequence of mutated *Us11* gene of HSV-1 strain**

GCGACCCAGA TGTTTACTTA AAAGGCGTGC CGTCCGCCGG CATGCACCCC AGAGGTGTTC

ACGCACCTCG AGGACACCCG CACATGATCT CCGGATCCCC GCAACGGGGT GATAATGATC

AAGCGGCGGG GCAATGTGGA GATTCGGGTC TACTACGAGT CCCCCGGGAG CCCCGGCCTC

CCCGGGAGCC CCGGACCCCA CGCACCCCCC GCAAACCACG TACGGCTCGC GGGTCTGTAT

AGCCCGGGCA AGTATGCCCC CCTGGCGAGC CCAGACCCCT TCTCCCCACA AGATGCAGCG

TACGCTCGGG CCCGCGTCGG GATCCACACC GCGGTTCGCG TTCCGCCCAC CGGAAGCCCA

ACCCACACGC ACTTGCGGCA CGACCCGGGC GATGAGCCAA CCTCGGATGA CTCAGGGCTC

TACCCTCTGG ACGCCCGGGC GCTTGCGCAC CTGGTGATGT TGCCCGCGGA CCACCGGGCC

TTCTTTCGAA CCGTGGTCGA GGTGTCTCGC ATGTGCGCTG CAAACGTGCG CGATCCCCCG

CCCCCGGCTA CAGGGGCCAT GTTGGGCCGC CACGCGCGGC TGGTCCACAC CCAGTGGCTC

CGGGCCAACC AAGAGACGTC GCCCCTGTGG CCCTGGCGGA CGGCGGCCAT TAACTTTATC

ACCACCATGG CCCCCCGCGT CCAAACCCAC CGACACATGC
