# Supplementary material for: A HSV1 mutant leads to an attenuated phenotype and induces immunity with a protective effect
Source: PLoS Pathog. 2020 Aug 10;16(8):e1008703. doi: 10.1371/journal.ppat.1008703 (PMC7440667; doi:10.1371/journal.ppat.1008703)
Supplement: S2 Text — Supplement to Fig 1 to display the mutated sequencing details of theUs12 gene of the M6 strain. (DOCX) [file ppat.1008703.s002.docx]

**S2 Text. The DNA sequence of mutated *Us12* gene of HSV-1 strain**

GAAATGGCGG ACACCTTCCT GGACAACATG CGGGTTGGGC CCAGGACGTA CGCCGACGTA

CGCGATGAGA TCAATAAAAG GGGGGCGTGA GGACCGGGAG GCGGCCAGAA CCCGCCGTGC

ACGACCCGGA GCGTCCCCTG CTGCGCTCTC CCGGGCTGCT GCCCGAAATC GCCCCCAACG

CATCCTTGGG TGTGGCACAT CGAAGAACCG GCGGGACCGT GACCGACAGT CCCCGTAATC

CGGTAACCCG TTGAGTCCCG GGTACGACCA TCGCCCGAGT TTCTGGGCGG AGGGTGGTTC

CCCCCGTGGC TCTCGAGATG AGCCAGACCC AACCCCCGGC CCCAGTTGGG CCGGGCGACC

CAGATGTTTA CTTAAAAGGC GTGCCGTCCG CCGGCATGCA CCCCAGAGGT GTTCACGCAC

CTCGAGGACA CCCGCACATG ATCTCCGGAT CCCCGCAACG GGGTGATAAT GATCAAGCGG

CGGGGCAATG TGGAGATTCG GGTCTACTAC GAGTCCCCCG GGAGCCCCGG CCTCCCCGGG

AGCCCCGGAC CCCACGCACC CCCCGCAAAC CACGTACGGC TCGCGGGTCT GTATAGCCCG

GGCAAGTATG CCCCCCTGGC GAGCCCAGAC CCCTTCTCCC CACAAGATGC AGCGTACGCT

CGGGCCCGCG TCGGGATCCA CACCGCGGTT CGCGTTCCGC CCACCGGAAG CCCAACCCAC

ACGCACTTGC GGCACGACCC GGGCGATGAG CCAACCTCGG ATGACTCAGG GCTCTACCCT

CTGGACGCCC GGGCGCTTGC GCACCTGGTG ATGTTGCCCG CGGACCACCG GGCCTTCTTT

CGAACCGTGG TCGAGGTGTC TCGCATGTGC GCTGCAAACG TGCGCGATCC CCCGCCCCCG

GCTACAGGGG CCATGTTGGG CCGCCACGCG CGGCTGGTCC ACACCCAGTG GCTCCGGGCC

AACCAAGAGA CGTCGCCCCT GTGGCCCTGG CGGACGGCGG CCATTAACTT TATCACCACC

ATGGCCCCCC GCGTCCAAAC CCACCGACAC ATGC
