# Supplementary figures and images for: A HSV1 mutant leads to an attenuated phenotype and induces immunity with a protective effect
Source: PLoS Pathog. 2020 Aug 10;16(8):e1008703. doi: 10.1371/journal.ppat.1008703 (PMC7440667; doi:10.1371/journal.ppat.1008703)

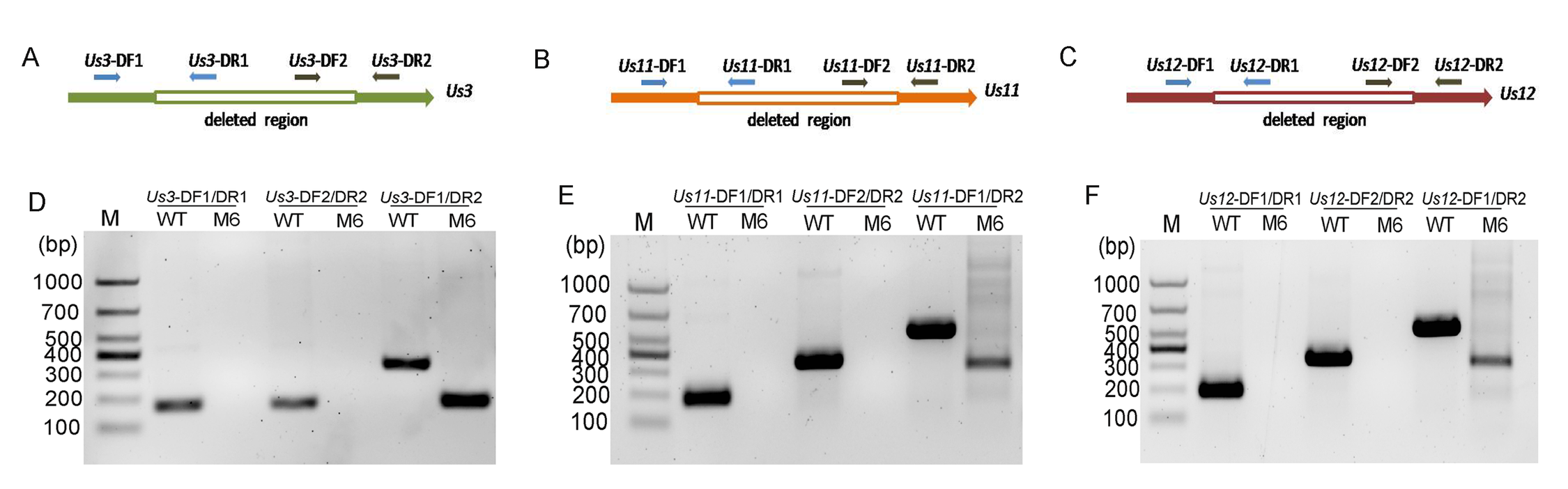

Supplement: S1 Fig — (A-C) Schematic for specific primer design of Us3(A), Us11(B) and Us12(C) genes. (D-F) PCR amplification of the M6 genome with specific primer pairs targeting the Us3 (D), Us11(E) and Us12(F) genes for verification of gene knockout. (TIF) [file ppat.1008703.s005.tif]

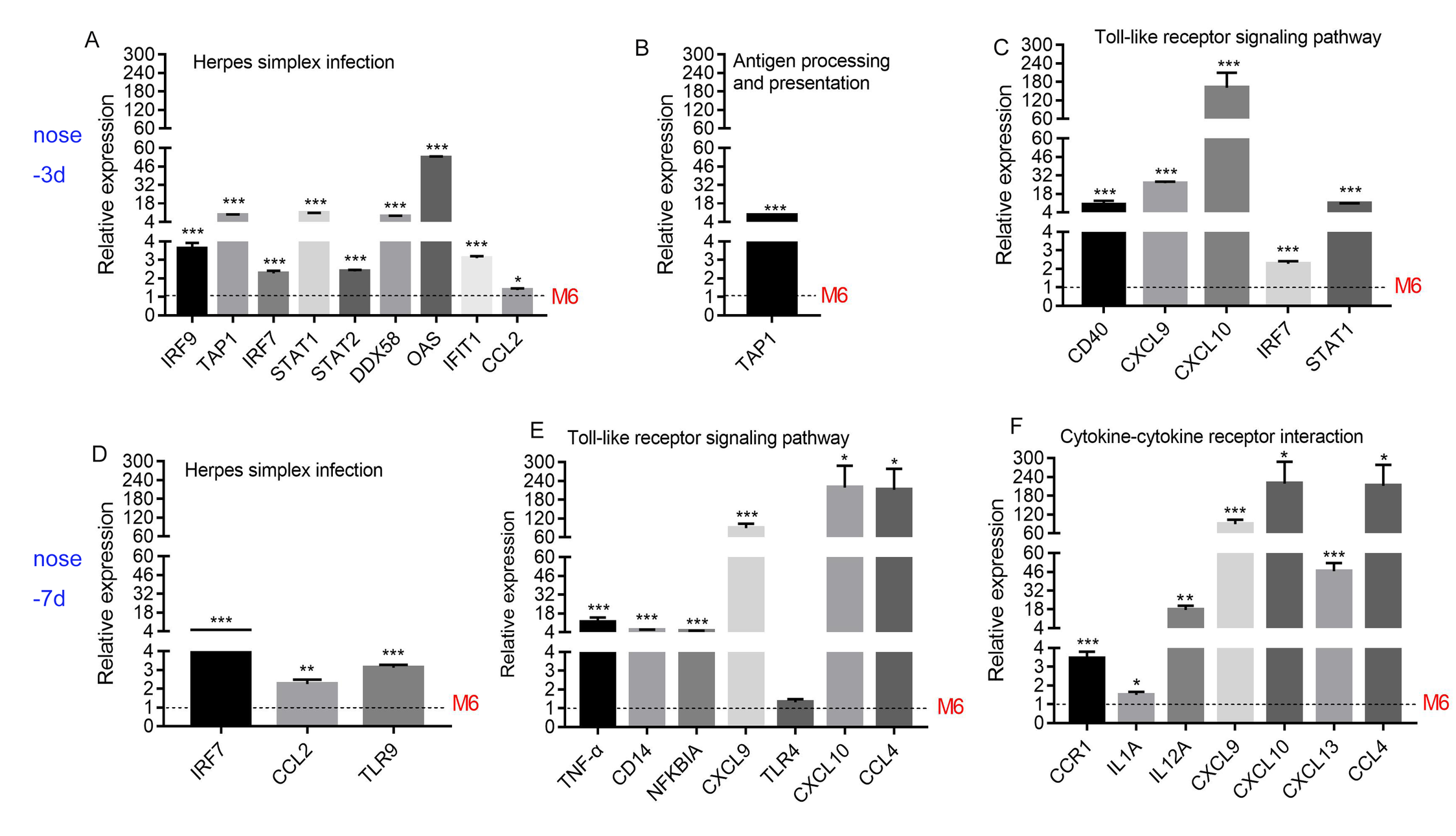

Supplement: S2 Fig — The nose tissues were collected at 1,3,7 days after viral infection followed by RNA extraction and q-RT-PCR assays. There was no difference at 1 d.p.i. (A-C) The gene expression of RNA samples at 3 d.p.i.(D-F)The gene expression of RNA samples at 7 d.p.i. Relative expression was measured by q-RT-PCR, and the column shows relative fold-change in expression compared with M6 (value = 1). mRNA levels were normalized using the geometric mean of GAPDH. The data are shown as the mean ± SEM based on data from three independent mice. *p< 0.05; **p< 0.01;*** p< 0.001. (TIF) [file ppat.1008703.s006.tif]

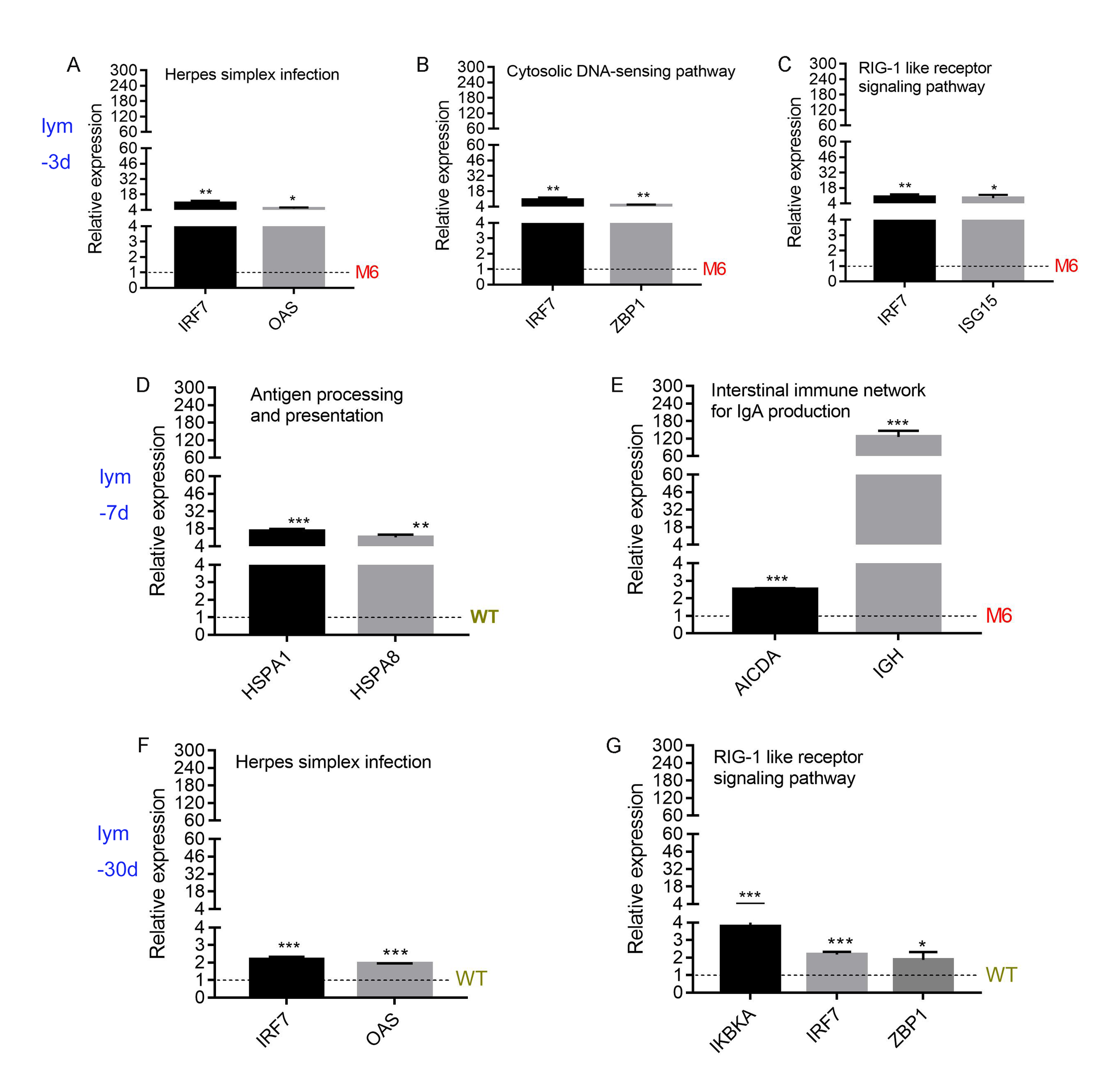

Supplement: S3 Fig — The splenic lymphocytes were collected at 3,7 and 30 days after viral infection followed by RNA extraction and q-RT-PCR assays.(A-C)The gene expression of RNA samples at 3d.p.i.(D,E)The gene expression of RNA samples at 7 d.p.i. (F,G)The gene expression of RNA samples at 30 d.p.i. Relative expression was measured by q-RT-PCR, and the column shows the relative fold-change in expression compared with M6 or WT (value = 1). The mRNA levels were normalized using the geometric mean of GAPDH. The data are shown as the mean ± SEM based on data from three independent mice. *p<0.05; **p< 0.01;***p<0.001. (TIF) [file ppat.1008703.s007.tif]
