# Supplementary material for: A HSV1 mutant leads to an attenuated phenotype and induces immunity with a protective effect
Source: PLoS Pathog. 2020 Aug 10;16(8):e1008703. doi: 10.1371/journal.ppat.1008703 (PMC7440667; doi:10.1371/journal.ppat.1008703)
Supplement: Attachment — Submitted filename: response.pdf [file ppat.1008703.s010.pdf]

The following is a point-to-point response to the reviewers' comments.

Part I - Summary

Reviewer #1: The manuscript # PPATHOGENS-D-19-02372 entitled "The assembly of mutations in 6 genes of HSV1 leads to an attenuated phenotype and induces immunity with a protective effect against viral infection in mice and rhesus macaques" constructed an HSV-1 vaccine candidate M6 with mutations in the UL7, UL41, LAT, Us3, Us11, Us12 genes. The M6 HSV-1 replicates at lower rate and have a small plaque phenotype in culture cells, and an attenuated phenotype in mouse and rhesus macaques. Authors also state that when immunizing mice and monkeys with the mutant strain M6, it induces remarkable serum neutralizing antibody titers and T cells activation, and protect against HSV1 challenge by impeding viral replication, dissemination and pathogenesis.

The significant weekness of this study is that while M6 induces neutralizing antibodies, and reduce the viral replication, the level of neutralizing antibody titer is very low, and M6 does not prevent virus replication in the tissues, and latency establishment in sensory ganglia. The concludion drawn from this study is not in the accordence with the data presented.

R: Thank you for the summary and comments. In our revised manuscript, we have added some experiments. The immunogenicity of M6 was evaluated in rhesus macaques via detection of their neutralizing antibody response and T cell response in a dose-dependent immunization test via the intramuscular route (Fig 5A).The results showed that immunizing monkey with three doses can induce significant proliferation of CD4+ and CD8+ T cells with IFN-γ specificity (Figs 5B and 5C). However, the neutralizing antibody titers showed a low titer until 28 d.p.i. (Fig 5D).

Then, the immunogenicity and protective effect of M6 were further investigated in a challenge test with the medium dose (Fig 6A). Although the neutralizing antibody titer is still low, theseimmunized animals were asymptomaticafter viral challenge, while all of the animals in the positive control group developed oral vesicles or exhibited redness around the eyes after WT strain challenge (Fig 6C). Histopathological detection also indicated the protectiveefficiency of M6.

For the latency establishment, we have no related experiments in this manuscript. However, our previous paper (Viruses. 2018 May 2;10(5). pii: E234.) has showed monkeys immunized with M3 could reduce the latency infection. M6 mutant is constructed based on the M3 strain, and three genes related with immunity have been modified. Thus, we think M6 could similarly reduce the latent infection, and we may perform more experiments to explore the latency establishment about M6-immunized monkey in our future work.

Reviewer #2: In this article, the authors established M6 mutants with mutations in the UL7, UL41, LAT, Us3, Us11, Us12 genes as an experimental attenuated vaccine against HSV1. The mutant exhibited attenuated phenotype in an animal model. Furthermore, in mice and rhesus monkeys, the mutant enables to induce remarkable serum neutralizing antibody titers and T cell activation and protect against HSV1 challenge. However, there are some issues to be addressed before further consideration of this ms

R: Thank you for the summary. We have added the experiments according to your suggestions.

Reviewer #3: In this study, the authors constructed an attenuated HSV1 virus strain with mutations in the UL7, UL41, LAT, Us3, Us11, Us12 genes. The animal models infected by the mutant virus, including mice and rhesus monkeys, had a remarkable T cell activation and neutralizing antibody. Moreover, the authors detected the proliferation characteristics and virulence phenotype in different cells and animal models. Overall, the study is important and interesting. However, some more experiments need to be done to strengthen their findings and conclusions.

R: Thank you for the summary. We have added the experiments to furtherstrengthen their findings and conclusionsaccording to your suggestions.

Part II- Major Issues: Key Experiments Required for Acceptance

Reviewer #1: No new experiments are suggested, because additional experiment will not make this manuscript better.

Reviewer #2: 1. In addition to detecting neutral antibodies and responsive T cell proliferation in mice or rhesus monkeys, CD4+ and CD8+ T cell responses and of cytokines expression should also be detected by ***flow cytometry****.*

R: This suggestion is very helpful for us. In the newly added dose-dependent inoculation experiment, CD4+ and CD8+ T cell responses and IFN-γ expression were detected by flow cytometry at 7, 14 and 28 days post immunization. The results showed that M6 immunization could induce the significant increase the numbers of $CD4^{+}$T IFN-γ^{+} and $CD8^{+}$T IFN-γ^{+} in both the mouse and rhesus monkey models. We have added these results to the Results section. These results are similar to the ELISpot results we used in the manuscript, which indicated that ELISpot assay is another available method for detecting the level of IFN-γ-producing lymphocytes.

2. The authors should take M6 inoculation in mice and rhesus monkeys in a dose-dependent manner to examine indicators of specific immune responses.

R: Thank youfor this useful suggestion. We inoculated the mice with three doses ($2x10^3$, $1x10^4$ or $5x10^4$pfu) and the rhesus monkeys with three doses ($2x10^4$, $1x10^5$ or $5x10^5$ CCID50) of M6. Moreover, we detected the neutralizing antibodies and responsive T cell proliferation in mice or rhesus monkeys. The results indicate that M6 immunization in three doses could induce a significant increase in the numbers of CD4+T IFN-γ+ and CD8+T IFN-γ+ in both the mouse and rhesus monkey models at 7, 14 and 28 days post immunization. However, at 28 days post immunization, we detectedonly low neutralizing antibody titers in the mouse and rhesus monkey models. We have added these results to the Results section.

3. To better reflect the reliability and authenticity of the research results, the authors should extend the experimental period and continuously observe the changes in neutralizing antibody levels and viral loads.

R: We think this issue is also very important for indicating the immunoprotectiveefficacy. Actually, we had previously reserved some of the monkeys for long-term immune response and protective observation. At this time point, three monkeys in M6-immunization or positive control groups were sacrificed followed by neutralizing antibody titer, viral load and pathological examination. The results suggested that the neutralizing antibody titer of M6-immunized monkeys was approximately 1:8. Compared with slight inflammatory cell infiltration in the positive control group monkeys, no viral load and inflammatory cell infiltration were found in the M6-immunized monkeys. We have added these results to the Results section.

Reviewer #3: 1.In Figure 1, the authors constructed an HSV1 strain with the assembly of mutations in 6 genes. However, what are the copies of HSV1 genome in every single cell and how the authors make sure that the Us3, Us11 and Us12 genes knockout in all HSV1 genomes.

R: The work of Lin C et al (Sci Rep. 2016 Oct 7;6:34531.) provided the first genetic evidence that two copies of the ICP0 gene in different locations on the same HSV-1 genome could be simultaneously modified with high efficiency and with no off-target modifications. In our manuscript, we designed specific primers surrounding and inside the mutated regions of the*Us3, Us11* and *Us12* genes. The PCR assays using

these primers indicated that the double copies of the*Us3, Us11* and *Us12* genes were modified (S1 Fig). We have added these results to the Results section.

2.Compared with an HSV-1 mutant vaccine with a UL18 deletion (ActaVirol. 2018;62(2):164-71), what advantages and advances of the M6 mutant vaccine has in cells and animal models.

R: First, the HSV-1 mutant vaccine with a UL18 deletion is a DNA vaccine, whereas the M6 mutant is alive attenuated vaccine; they are different vaccine types. Second, in the cell assay, UL18 deletion vaccine failed to achieve rescue to indicate the safety of the DNA in cell culture. In our experiment, the M6 mutant showed low viral proliferation capacity in the cell culture. Finally, in the animal models, the UL18 deletion vaccine could induce specific T-lymphocyte proliferation responses and a very low neutralizing antibody level. Correspondingly, the M6 mutant could induce both T cell response proliferation and a high neutralizing antibody level. Moreover, there are no data to analyze the immunoprotectiveeffectsof the UL18 deletion vaccine.

Part III- Minor Issues: Editorial and Data Presentation Modifications

Reviewer #1: 1.The title of this manuscript suggests that M6 mutant induces immunity with a protective effect against viral infection in mice and rhesus macaques; however, the data clearly shows that it does not prevents viral infection in mice or rhesus.

R: Based on the results in mice and rhesus monkeys, the M6 mutant induces remarkable serum neutralizing antibody titers and T cell activation and protects against HSV1 challenge by impeding viral replication, dissemination and pathogenesis. Thus, we can state that the M6 mutant can prevent viral infection in mice or rhesus monkeys.

2. Many sections of figures lack the titles or the details on the y axis.

R: We have modified the figures by adding more details tothe *y-axis.*

3. In the introduction, authors stated that HSV-1 cause occasional genital herpes

infection. Epidemiology indicates that more that 50% of first-time genital herpes infections are now caused by HSV-1 virus.

R: We have checked the relevant reference and revised the sentences in the introduction section.

Reviewer #2: None

Reviewer #3: 1.In Figure 1C-G, the authors detected biological properties such as plaques number, growth curves of the M6 strain, M3 strain and WT strain. The authors could also observe the M3 infection of Balb/c mice to further strengthen their conclusions that a more significantly attenuated phenotype for the M6 strain than for the WT strain.

R: We have added this experiment to our manuscript. The results showed that the viral growths in nasal and spleen tissues of M6-infected mice were significantly lower than in M3-infected mice.

2.In Figure 3, the authors utilized omic analysis of mRNA expression to suggest a characteristic immune response initiated by M6. However, the conclusions seem unconvincingly unless the authors determine the gene expression by qPCR and ELISA.

R: Thank youfor this useful suggestion. According to the suggestion, we have designed many primers specific to the pathways. Quantitative RT-PCR (qRT-PCR) assay was used to analyze and confirm the omics analysis results of the gene expression measured by the ΔCt value (S2 and S3 Figs).

3.In Figure 4, to confirm their hypothesis that mutation of some virally encoded proteins with functions inhibiting different key molecules in the innate and adaptive immune responses could lead to the host immune system developing an integrated response against viral antigens, the authors should apply flow cytometry technology to detect the T cell activation.

R:In the revised manuscript, we have added a dose-dependent inoculation experiment. The CD4+ and CD8+ T cell responses and IFN-γ expression were detected by flow cytometry at 7, 14 and 28 days post immunization. The results

showed that M6 immunization could induce a significant increase in the numbers of $CD4^+T$ IFN-γ^+ and $CD8^+T$ IFN-γ^+ in both the mouse and rhesus monkey models. We have added these results to the Results section. These results are similar to the ELISpot results we used in the manuscript, which indicated that ELISpot assay is also an available method for detecting the level of IFN-γ producing lymphocytes.

4.In Figure 4 and Table 3, the authors demonstrated that there is no difference between M3 - and M6-immunized mice, suggesting that M3 strain is enough for inducing specific neutralizing antibody and CTL responses. Therefore, compared with M3 strain, please clarify what are the advantages and improvements of M6 strain.

R:In our other experiments (unpublished data), we confirmed using two-dimensional electrophoresis (2-DE) and mass spectrometry that the serum antibodies of HSV1-positive patients mainly recognized capsid proteins, such as proteins encoded by UL26.5 and UL42 genes. Meanwhile, the membrane structural glycoproteins, which are the main immunogens recognized by neutralizing antibodies, were not detected. It is suggested that these encoded capsid proteins are likely to be the dominant antigens that interact with immune response and stimulate the immune system to produce antibodies during the HSV1 infection. Based on these results, we think that the neutralizing antibody titer cannot be used as aunique factor to evaluate viral immunogenicity. In our manuscript, there was no difference in neutralizing antibody titers between M3- and M6-immunized mice;however, we modified more genes involved in the apoptosis and antigen presentation of the M6 mutant, and M6 may be safer than the M3 strain.
