# Supplementary material for: A HSV1 mutant leads to an attenuated phenotype and induces immunity with a protective effect
Source: PLoS Pathog. 2020 Aug 10;16(8):e1008703. doi: 10.1371/journal.ppat.1008703 (PMC7440667; doi:10.1371/journal.ppat.1008703)
Supplement: Attachment — Submitted filename: Review ppathogens-D-19-02372 2-14-20.docx [file ppat.1008703.s008.docx]

Major: The manuscript # PPATHOGENS-D-19-02372 entitled “The assembly of mutations in 6 genes of HSV1 leads to an attenuated phenotype and induces immunity with a protective effect against viral infection in mice and rhesus macaques” constructed an HSV-1 vaccine candidate M6 with mutations in the UL7, UL41, LAT, Us3, Us11, Us12 genes. The M6 HSV-1 replicates at lower rate and have a small plaque phenotype in culture cells, and an attenuated phenotype in mouse and rhesus macaques. Authors also state that when immunizing mice and monkeys with the mutant strain M6, it induces remarkable serum neutralizing antibody titers and T cells activation, and protect against HSV1 challenge by impeding viral replication, dissemination and pathogenesis.

While M6 induces neutralizing antibodies, and reduce the viral replication, the level of neutralizing antibody titer is very low, and M6 does not prevent virus replication in the tissues, and latency establishment in sensory ganglia.

The title of this manuscript suggests that M6 mutant induces immunity with a protective effect against viral infection in mice and rhesus macaques; however, the data clearly shows that it does not prevents viral infection in mice or rhesus.

Many sections of figures lack the titles or the details on the y axis.

In the introduction, authors stated that HSV-1 cause occasional genital herpes infection. Epidemiology indicates that more that 50% of first-time genital herpes infections are now caused by HSV-1 virus.
