# Supplementary material for: A HSV1 mutant leads to an attenuated phenotype and induces immunity with a protective effect
Source: PLoS Pathog. 2020 Aug 10;16(8):e1008703. doi: 10.1371/journal.ppat.1008703 (PMC7440667; doi:10.1371/journal.ppat.1008703)
Supplement: Attachment — Submitted filename: Review.doc [file ppat.1008703.s009.doc]

In this study, the authors constructed an attenuated HSV1 virus strain with mutations in the UL7, UL41, LAT, Us3, Us11, Us12 genes. The animal models infected by the mutant virus, including mice and rhesus monkeys, had a remarkable T cell activation and neutralizing antibody. Moreover, the authors detected the proliferation characteristics and virulence phenotype in different cells and animal models. Overall, the study is important and interesting. However, some more experiments need to be done to strengthen their findings and conclusions.

Major points:

1. In Figure 1, the authors constructed an HSV1 strain with the assembly of mutations in 6 genes. However, what are the copies of HSV1 genome in every single cell and how the authors make sure that the Us3, Us11 and Us12 genes knockout in all HSV1 genomes.
2. Compared with an HSV-1 mutant vaccine with a UL18 deletion (Acta Virol. 2018;62(2):164-71), what advantages and advances of the M6 mutant vaccine has in cells and animal models.

Minor points:

1. In Figure 1C-G, the authors detected biological properties such as plaques number, growth curves of the M6 strain, M3 strain and WT strain. The authors could also observe the M3 infection of Balb/c mice to further strengthen their conclusions that a more significantly attenuated phenotype for the M6 strain than for the WT strain.
2. In Figure 3, the authors utilized omic analysis of mRNA expression to suggest a characteristic immune response initiated by M6. However, the conclusions seem unconvincingly unless the authors determine the gene expression by qPCR and ELISA.
3. In Figure 4, to confirm their hypothesis that mutation of some virally encoded proteins with functions inhibiting different key molecules in the innate and adaptive immune responses could lead to the host immune system developing an integrated response against viral antigens, the authors should apply flow cytometry technology to detect the T cell activation.
4. In Figure 4 and Table 3, the authors demonstrated that there is no difference between M3 - and M6-immunized mice, suggesting that M3 strain is enough for inducing specific neutralizing antibody and CTL responses. Therefore, compared with M3 strain, please clarify what are the advantages and improvements of M6 strain.
