# Supplementary material for: A HSV1 mutant leads to an attenuated phenotype and induces immunity with a protective effect
Source: PLoS Pathog. 2020 Aug 10;16(8):e1008703. doi: 10.1371/journal.ppat.1008703 (PMC7440667; doi:10.1371/journal.ppat.1008703)
Supplement: Attachment — Submitted filename: Review ppathogens-D-19-02372R1 6-4-20 .docx [file ppat.1008703.s011.docx]

The revised manuscript # PPATHOGENS-D-19-02372R1 entitled “The assembly of mutations in 6 genes of HSV1 leads to an attenuated phenotype and induces immunity with a protective effect against viral infection in mice and rhesus macaques” designed an HSV-1 vaccine candidate M6 with mutations in the UL7, UL41, LAT, Us3, Us11, Us12 genes. Immunizing mice and monkeys with the mutant strain M6, neutralizing antibody titers and T cells activation, and protect against HSV1 disease.

The data presented in the study show M6 vaccine can prevent mice and rhesus from the HSV-1 disease (Figure 6C). Measurement of virus replication in eye swabs in HSV-1 challenged rhesus, both mock and vaccinated group showed similar level of viral copies (Figure 6F). The data show that vaccine can prevent disease but not the infection. As a matter of fact, the most vaccines against infectious diseases do not prevent infection, however they prevent disease. I propose the title and conclusion of this study should correctly reflect that. Title of this manuscript could be modified to “The assembly of mutations in 6 genes of HSV1 leads to an attenuated phenotype and induces protective immunity and prevent HSV-1 disease in mice and rhesus macaques.” The conclusion should also be revised accordingly.
